# La protein regulates protein expression by binding with the mRNAs of target genes and participates the pathological process of ovarian cancer

**DOI:** 10.3389/fonc.2022.763480

**Published:** 2022-08-30

**Authors:** Xuan Huang, Jialei Zhu, Yueyan Li, Yang Yu, Jing Tang

**Affiliations:** Department of Pharmacy, Obstetrics and Gynecology Hospital of Fudan University, Shanghai, China

**Keywords:** La protein, ovarian cancer, apoptosis, quantitative proteomics, immunoprecipitation

## Abstract

Research on the mechanism and new targets of ovarian cancer is of great significance to reduce the high mortality and drug resistance of ovarian cancer. Human La protein has been found to be highly expressed in a variety of malignant tumors and plays a role in tumorigenesis and development through its RNA-binding function. However, its role and mechanism in ovarian cancer are not completely clear. The present study showed that La protein was highly expressed in serum and tissues of patients with ovarian cancer by ELISA and immunohistochemistry, and the high expression of La protein was associated with the increased degree of malignancy and poor prognosis by searching the KM plotter database. Interference of the La gene resulted in a significant decrease in the proliferation, migration, and invasion of ovarian cancer cells with growth block in the G1 phase and increasing apoptosis. By RNA binding protein immunoprecipitation, transcriptome sequencing, and proteomics, 14 downstream target genes were screened. The La protein might affect the protein expression of these 14 genes by binding with the mRNAs. Therefore, it played a role in the pathological process of ovarian cancer.

## Introduction

Among all gynecological cancer-related deaths, ovarian cancer has the highest mortality rate. Approximately 239,000 new cases of ovarian cancer are diagnosed each year, of which 152,000 die of the disease (1). In developed countries, ovarian cancer is the second most common malignancy in women over 40 years old, and its incidence rate is second only to breast cancer (2). For women, the incidence rate of ovarian cancer ranks 11th in all cancers, but the mortality rate ranks fifth among all cancers (3). Although great progress has been made in the treatment of ovarian cancer in recent years, the long-term cure rate has not changed significantly in the past 20 years, and the 5-year survival rate is only 47.4% (3). Chemotherapy resistance is still an important cause of high mortality of ovarian cancer. Therefore, it is of great significance to further study the pathogenesis of ovarian cancer and find new therapeutic targets for it.

La protein, also known as Sjögren’s syndrome antigen B (SSB), is a multifunctional RNA-binding protein, which plays an important role in RNA processing and metabolism (4). It can specifically bind to the terminal motif UUU-3′OH of the RNA polymerase III (RNAP III) transcript to promote RNA biosynthesis (5) and protects them from exonuclease decay. La protein promotes the production of tRNA, processes the 5-prime and 3-prime terminals of the pre-tRNA precursor, and acts as an RNA chaperone. It combines with specific RNA molecules to regulate its downstream processing. La protein promotes the correct folding of transcripts, prevents the misfolding, and promotes the maturation of transcripts (6, 7). It also participates in the internal ribosome entry site (IRES)-mediated translation of mRNA (8, 9).

La protein is highly expressed in various malignant tumors, and its expression is related to the clinical TNM stage, lymph node metastasis, and differentiation degree of tumor cells, which plays an important role in the translation control of tumor promoters (10–13). It can promote the tumorigenicity, change the cell cycle, and promote the proliferation, migration, and invasion of cancer cells (12, 14, 15). It can also promote the translation of Bcl-2, which may promote drug resistance by inhibiting the apoptosis of cancer cells. La protein mediates transforming growth factor β (TGF β)-induced epithelial–mesenchymal transition and cancer stem cell characteristics, contributing to the proliferation, migration, chemoresistance, and tumor growth of mouse cancer cells (10). It is an essential component of the X-linked inhibitor of apoptosis protein (XIAP) IRES ribonucleoprotein complex. La protein specifically binds to XIAP IRES and interacts with XIAP IRES to form the core of the ribonucleoprotein complex. It enhances the IRES-dependent translation of XIAP and upregulates the expression of XIAP (16). The increase in La protein content can directly stimulate the translation of β-catenin mRNA or stimulate the positive regulators of β-catenin (17). It can activate the translation of MDM2 mRNA by binding 5′UTR to murine double minute 2 (MDM2) mRNA (18). La protein plays an important role in malignant tumors by regulating the posttranscriptional translation of these genes.

In the present study, we detected the levels of the La protein in serum and tissues of patients with ovarian cancer by ELISA to evaluate the relationship between La protein and the high degree of malignancy. We confirmed the results by searching the KM plotter database. Then we explored the mechanisms of La protein in ovarian cancer by interference of the La gene. Furthermore, we did the proteomic analysis of A2780 cells to screen target genes. These findings illustrated that La protein played its RNA chaperone function by binding with the mRNA of target genes and had a role in the pathological process of ovarian cancer. It provided a new strategy for the treatment of ovarian cancer.

## Materials and methods

### Survival analysis

The Kaplan–Meier plotter database (KM plotter) (https://kmplot.com/analysis/) was used to integrate gene expression and clinical data simultaneously. To determine the prognostic value of La, patients were split by auto-selecting the best cutoff, and hazard ratios with 95% confidence interval and log-rank P value were calculated.

### Clinical samples

The study subjects, including 12 healthy volunteers and 17 cases of ovarian cancer, were enrolled between March 2019 and May 2019 at the Obstetrics & Gynecology Hospital of Fudan University. The whole blood sample from each subject was collected in the anticoagulant tube for analysis of the La protein level. The blood sample collection and analysis protocols were approved by the ethics committee of the Obstetrics & Gynecology Hospital of Fudan University (No. 2019-35).

Ovarian cancer tissue microarray (cat. no. OVC1021) was brought from Fanpu Biotech, Inc., which contains two cases of normal ovarian tissue, three cases of ovarian cystadenoma tissues, one case of ovarian clear cell adenocarcinoma (OCCA), 48 cases of ovarian serous cystadenocarcinoma tissues (OSC), 30 cases of ovarian endometrioid adenocarcinoma (OEA) tissues, and 16 cases of ovarian mucinous cystadenocarcinoma (OMC) tissues.

### Elisa

Two milliliters of whole-blood sample collected from each subject was centrifugated at 500 g for 5 min to obtain the serum. Then, the serum La protein concentration was evaluated with Human Sjogren’s syndrome antigen B (SSB) ELISA kit from Shanghai Enzyme-linked Biotechnology Company according to the standard procedure of ELISA assay. Each sample was set to two wells. The optical density (OD) value of each well was measured at a wavelength of 450 nm.

### Cell culture

Human EOC cell lines (OVCAR3, SKOV3, and A2780) were obtained from Shanghai Genechem Co., Ltd. (Shanghai, China). The normal human ovarian epithelial cell IOSE80 was bought from Yuchicell (Shanghai) Biological Technology Co., Ltd. (Shanghai, China). Cells were cultivated in RPMI 1640 (HyClone Laboratories, Logan, UT, USA) medium, supplemented with 10% fetal bovine serum (FBS) (Gibco, Grand Island, NY, USA), 2 mM L-glutamine, 100 U/ml penicillin, and 100 U/ml streptomycin (all from (Biological Industries, Beit HaEmek, Israel) at 37°C and 5% CO_2_. All the stable transgenic cell lines were maintained below five passages.

### Short hairpin RNA transduction

Three predesigned La-interference shRNA lentivirus particles were purchased from Genechem (Shanghai, China): LV-SSB-RNAi (89851-21) # (5′-ccTGCATCCAAACAACAGAAA-3′), LV-SSB-RNAi (89852-21) # (5′-ccAACAAGAATCCCTAAACAA-3′), and LV-SSB-RNAi (89853-21) # (5′-gcTGAAATGAAATCTCTAGAA-3′), with the GV248 expression vector. The negative control lentivirus particle was also provided by Genechem (cat. no. LVCON077; sequence, 5′-TTCTCCGAACGTGTCACGT-3′; vector, GV248). Infected cell populations rather than single clones were selected by 2.5 μg/ml puromycin for 2 weeks to generate stable cell lines. The expression of La was detected by Western blot assay and real-time PCR to demonstrates that the knockdown was successful.

### Cell viability assay

Cell viability was analyzed by Cell Counting Kit-8 (CCK-8, Dojindo, Kumamoto, Japan) according to the manufacturer’s protocols. Two thousand cells/well were seeded into 96-well plates. Ten microliters of CCK-8 reagent was added to detect cell proliferation at 1–5 days by reading the optical density value at a wavelength of 450 nm at 4 h after WST-8 incubation. All experiments were performed in triplicate.

### Western blotting assay

Cells were collected and proteins were extracted using RIPA buffer containing 1 mmol/l PMSF (Beyotime Biotechnology, Shanghai, China), and the concentration of total protein or protein after co-immunoprecipitation was quantified using the BCA Protein Assay Kit (Beyotime Biotechnology, Shanghai, China). An equal amount of proteins was separated by SDS‐PAGE on 10% gel prepared using PAGE Gel Fast Preparation Kit (cat. no. PG112, Epizyme, Shanghai, China). Moreover, proteins were transferred to PVDF membranes (Millipore, Massachusetts, USA), after blocking with 5% defatted milk powder in phosphate‐buffered saline with Tween (PBST) for 2 h. Membranes were then incubated with primary anti-La antibody (1:10,000; cat. no. ab75927; Abcam), anti-PKM antibody (1:10,000; cat. no. ab150377; Abcam), anti-NQO1 antibody (1:25,000; cat. no. ab80588; Abcam), anti-IGF2BP1 antibody (1:1,000; cat. no. ab184305; Abcam), anti-TK1 antibody (1:1,000; cat. no. ab76495; Abcam), anti-Calreticulin antibody (1:1,000; cat. no. #12238T; Cell Signaling Technology), anti-CD9 antibody (1:1,000; cat. no. ab263019; Abcam), anti-Sall4 antibody (1:1,000; cat. no. #8459; Cell Signaling Technology), anti-HLAB antibody (1:1,000; cat. no. ab193415; Abcam), anti-SLC2A1 antibody (1:50,000; cat. no. ab115730; Abcam), anti-Bad antibody (1:1,000; cat. no. ab32445; Abcam), anti-MAP2K1 antibody (1:1,000; cat. no. ab32091; Abcam), anti-Rif1 antibody (1:500; cat. no. ab229632; Abcam), Anti-Nestin antibody (1:1,000; cat. no. #10959s; Cell Signaling Technology), and anti-FHIT antibody (1:1,000; cat. no. ab170888; Abcam) followed by incubation with a secondary antibody (1:10,000; cat. no. #7074; Cell Signaling Technology). Anti-GAPDH antibody (1:2,000; cat. no. sc-32233; Santa Cruz) was used as a control.

### Transwell invasion and migration assay

The Transwell invasion and migration assays were performed using Transwell chambers (8-μm pores; Corning, Corning, NY, USA) coated with Matrigel or without Matrigel (Corning). For migration assays, 1 × 10^5^ cells suspended in 100 μl serum-free medium were seeded in the upper chamber. The bottom chamber was added with 600 μl medium containing 30% FBS as a chemoattractant. For invasion assays, 1 × 10^5^ cells/well in 200 μl serum-free medium were added to the upper chamber and 750 μl 30% FBS serum medium was added to the lower chamber. After 24 h of incubation for migration and 48 h for invasion, non-migrating cells were removed by wiping the upper chamber. Migrated/invaded cells were stained with crystal violet and counted under a microscope. All experiments were performed in triplicate.

### DNA cell-cycle analysis

Cells were harvested, and a single-cell suspension was prepared in complete medium. Cells were washed and spun at 1,300 rpm for 5 min in D-Hanks solution. The cells were fixed with 75% ethanol precooled at 4°C for at least 1 h and washed by D-Hanks solution again. The cells were centrifuged and stained with propidium iodide staining buffer (2 mg/ml propidium iodide: 10 mg/ml RNase solution: D-Hanks solution = 25:10:1,000), followed by analysis by flow cytometry.

### Cell apoptosis

The experiment was conducted using ApoScreen Annexin V Apoptosis Kit (cat. no. 10010-09; AL, USA) and flow cytometry (version 10.0, FlowJo, FACSCalibur™, BD, USA) according to the manufacturer’s instructions. Briefly, 200 μl 1× binding buffer was added to suspend the cells, and 10 μl of Annexin V-PE was added and incubated for 15 min in the dark. Then, 10 µl 7-AAD and 400 μl binding buffer were added before flow cytometry.

### Real-time PCR

TRIzol reagent (TaKaRa, Kusatsu, Japan) was used for RNA extraction, followed by cDNA synthesis using M-MLV Reverse Transcriptase (cat. no. M1705; Promega Corporation, WI, USA). RT-qPCR primer assays and SYBR Master Mixture were purchased from TaKaRa. For each condition, triplicates were run, and data were normalized to the expression levels of housekeeping genes GAPDH *via* the comparative *C*_t_ method. The sequence of oligonucleotide primers La (forward primer: 5′-CCAACTGATGCAACTCTTGAT-3′ reverse primer: 5′-TTTTGGCAAAGTAATCGTCC-3′) and GAPDH (forward primer: 5′-TGACTTCAACAGCGACACCCA-3′ reverse primer: 5′-CACCCTGTTGCTGTAGCCAAA-3′) were synthesized by Sangon (Shanghai, China).

### Immunohistochemistry for tissue microarrays

Immunohistochemistry on tissue microarray was used to detect the expression of human La protein in ovarian cancer tissues. Arrays were dewaxed, rehydrated, submerged into EDTA buffer for antigen retrieval, and doused with 3% H_2_O_2_ to block endogenous peroxidase. After antigen blocking with 5% BSA, anti-La antibody (1:250; cat. no. ab75927; Abcam) was incubated at 4°C overnight, and the secondary antibody was incubated at room temperature for 30 min using SABC immunohistochemical staining kit (cat. no. SA1028; BOSTER, Wuhan, China). Antibody specificity was confirmed by a negative control without primary antibody treatment. The reaction was visualized using DAB, and the slides were counterstained with hematoxylin. The images were scanned and analyzed using Aperio ImageScope (Leica, Germany). The analysis software automatically identified all dark-brown tissue sections as strongly positive, brownish yellow as moderately positive, light yellow as weakly positive, and blue as negative. Histochemistry score (H-SCORE) = (percentage of weakly positive cells × 1) + (percentage of moderate positive cells × 2) + (percentage of positive cells × 3) (19).

### RIP-seq

RIP was performed using the EZ-Magna RIP™ RNA-Binding Protein Immunoprecipitation Kit (cat. no. 17-701; Millipore), according to the manufacturer’s protocol. Magnetic beads coated with 5 μg of specific anti-La antibody (cat. no. ab124932; Abcam), positive control Anti-SNRNP70 antibody (Millipore), or normal IgG (Millipore) were incubated with prepared indicated cell lysates overnight at 4°C. RNA–protein complexes washed with RIP wash buffer were treated with Proteinase K Buffer. The immunoprecipitated RNAs were purified with phenol: chloroform: isoamyl alcohol (125: 24: 1) and subsequently subjected to high-throughput sequencing. RNA purity was checked using the NanoDrop 2000 (Implen, CA, USA). Moreover, the RNA concentration was measured using Qubit^®^ RNA Assay Kit in Qubit^®^ 2.0 Fluorometer (Life Technologies, CA, USA). RNA integrity was assessed using the RNA Nano 6000 Assay Kit of the Agilent Bioanalyzer 2100 system (Agilent Technologies, CA, USA). Briefly, products were purified with the AMPure XP system (Beckman Coulter, Beverly, USA) and library quality was assessed on the Agilent Bioanalyzer 2100 system. The clustering of the index-coded samples was performed on a cBot Cluster Generation System using TruSeq PE Cluster Kit v3-cBot-HS (Illumina) according to the manufacturer’s instructions. After cluster generation, the libraries were sequenced on an Illumina HiSeq 4000 platform. The raw sequencing data from this study have been deposited in the Genome Sequence Archive for Human (https://ngdc.cncb.ac.cn/gsa-human/), under the accession number HRA002613.

### TMT labeling and MS analysis

After protein extraction, SDS-PAGE separation, and filter-aided sample preparation, 100 μg peptide mixture of each sample was labeled using TMT10plex™ Isobaric Mass Tagging Kit according to the manufacturer’s instructions (cat. no. 90113; Thermo Fisher Scientific, Waltham, MA, USA). TMT-labeled peptides were fractionated by reversed-phase (RP) chromatography using the Agilent 1260 Infinity II HPLC. Each fraction was injected for nano LC-MS/MS analysis. The peptide mixture was loaded onto the C18-reversed phase analytical column (cat. no. P/N164943; Thermo Fisher Scientific) in buffer A (0.1% formic acid) and separated with a linear gradient of buffer B (80% acetonitrile and 0.1% formic acid) at a flow rate of 300 nl/min. LC-MS/MS analysis was performed on a Q Exactive HF mass spectrometer (Thermo Fisher Scientific) that was coupled to Easy-nLC (Thermo Fisher Scientific) for 60 min. The mass spectrometry proteomics data have been deposited to the ProteomeXchange Consortium (http://proteomecentral.proteomexchange.org) *via* the iProX partner repository (20) with the dataset identifier PXD033569.

### Statistical analysis

Analyses were conducted using GraphPad Prism 8.0.2, and data were expressed as mean ± standard deviation (SD). Student’s *t-*test and one-way ANOVA were used for statistical analysis. p < 0.05 was considered statistically significant. All experiments were repeated three to five times.

## Results

### The high expression of La protein in ovarian cancer was related to the high degree of malignancy

**Figure 1:**
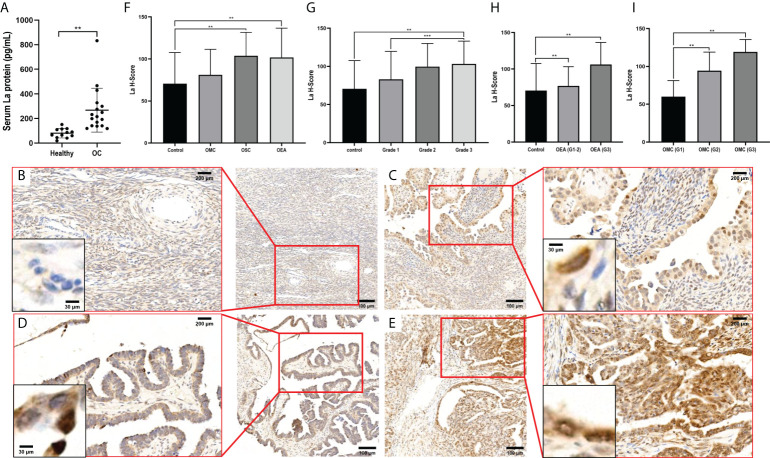
Expressions of La protein in ovarian cancer. **(A)** The expression of La protein in serum of healthy volunteers and patients with ovarian cancer. Typical images of La protein expression in different types of ovarian tissues: normal ovarian tissue **(B)**, ovarian serous cystadenocarcinoma tissue **(C)**, ovarian mucinous cystadenocarcinoma tissue **(D)**, and ovarian endometrioid adenocarcinoma tissue **(E)**. The expression of La protein in ovarian cancer **(F)** and its relationship with the pathological grade of ovarian cancer **(G)**. **(H)** The expression of La protein in ovarian endometrioid adenocarcinoma. **(I)** The expression of La protein in ovarian serous cystadenocarcinoma. The statistical methods were t-test and t-test one-way ANOVA. All data were expressed as mean ± SD (^**^ *P* < 0.05, ^***^ *P* < 0.01). OC, ovarian cancer; OEA, ovarian endometrioid adenocarcinoma; OMC, ovarian mucinous cystadenocarcinoma; OSC, ovarian serous cystadenocarcinoma tissues.

The expressions of La protein in serum of patients with ovarian cancer and healthy volunteers were detected by ELISA. As shown in **Figure 1A**, the expression of La protein in serum of patients with ovarian cancer was significantly higher than that of the control group (p = 0.019). The expressions of La protein in ovarian cancer tissues were further investigated by immunohistochemistry. It showed that the expression of La protein in serous cystadenoma and endometrial adenocarcinoma of ovarian cancer was significantly higher than that in normal and benign ovarian tissues (). No significant difference was detected between La protein expression in ovarian mucinous cystadenocarcinoma and that in normal and benign ovarian tissues. It may be due to the variation of La expression in clinical samples and the limited number of cases (**Figures 1B–F**). Moreover, the expression of La protein in ovarian mucinous cystadenocarcinoma tissues of each case classification was separately counted. It showed that the expression of La protein in ovarian mucinous cystadenocarcinoma tissue was statistically significantly related to the malignant degree of pathological grade. The results indicate that the expression of La protein is meaningful in mucinous ovarian cystadenocarcinoma. A high expression of La protein was also observed in ovarian endometrial adenocarcinoma, and the pathological grade of malignant degree was relatively increased. The results are shown in **Figures 1G–I**. These results indicated that the high expression of La protein in ovarian cancer was related to the high degree of malignancy.

### High expression of La in ovarian cancer predicts poor prognosis

**Figure 2:**
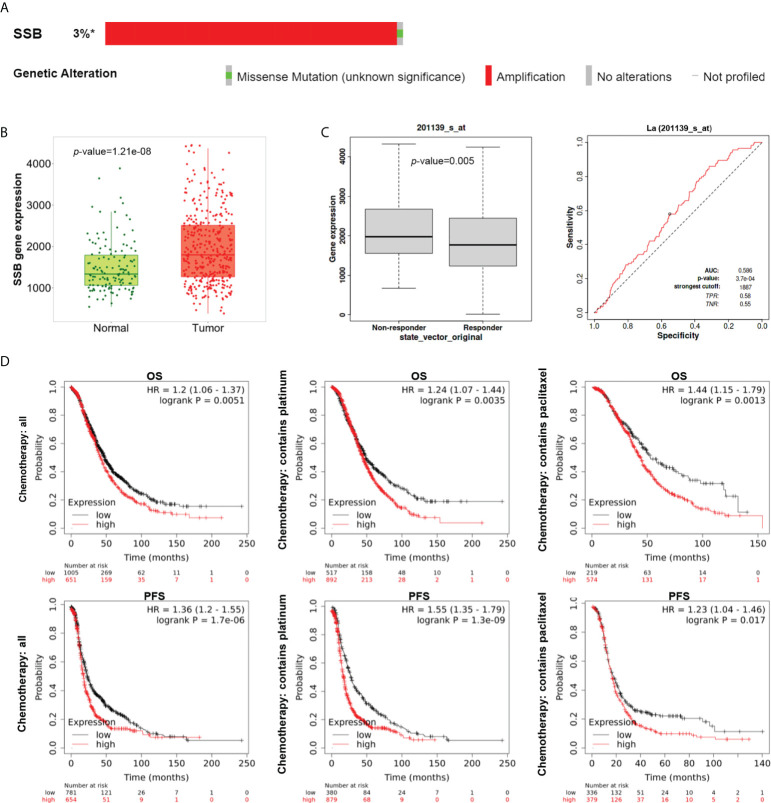
Expression, alternation, and prognostic analysis of La in ovarian cancer by bioinformatics analysis. **(A)** The alterations of the La gene in ovarian cancer (cBioPortal). La gene was examined in all five ovarian cancer studies with >3% gene alterations. **(B)** The mRNA expression pattern of La from TNMplot between ovarian cancer tissues (red) and normal samples (green) from non-cancerous patients and further pediatric tissues. **(C)** Boxplot and ROC curve of La involved in platinum resistance using the RFS at 6 months cohort from the ROC plotter for ovarian cancer (Affymetrix ID: 201139_s_at). **(D)** The prognostic value of mRNA level of La in ovarian cancer patients from the KM plotter (Affymetrix ID: 201139_s_at). ^*^, altered / profiled=49 / 1657.

Mutation analysis of the La gene in ovarian cancer patients by the cBioPortal database showed that the most common genetic alternation of La is amplification (**Figure 2A**). Using the TNMplot database, we analyzed the mRNA expression of La between ovarian cancer tissues and normal samples. The expression of La was higher in ovarian cancer tissues (**Figure 2B**), which is in accordance with the ELISA results above. We filtered for ovarian cancer patients who received platinum therapy in the ROC plotter database (21). Mann–Whitney test and ROC analysis were conducted, and results showed that the high expression of La is related to the poor response to platinum-based chemotherapy (**Figure 2C**). The KM plotter database was used to analyze the correlations between the expression of La protein and overall survival (OS) and progression-free survival (PFS) in ovarian cancer patients and ovarian cancer patients treated with platinum and paclitaxel. It showed that the OS and PFS of ovarian cancer patients with high expression of La were lower. The high expression of La is also significantly correlated with the poor OS and PFS of platinum and paclitaxel-based chemotherapy. These results are shown in **Figure 2D**.

### The expression of La protein was interfered by lentivirus

**Figure 3:**
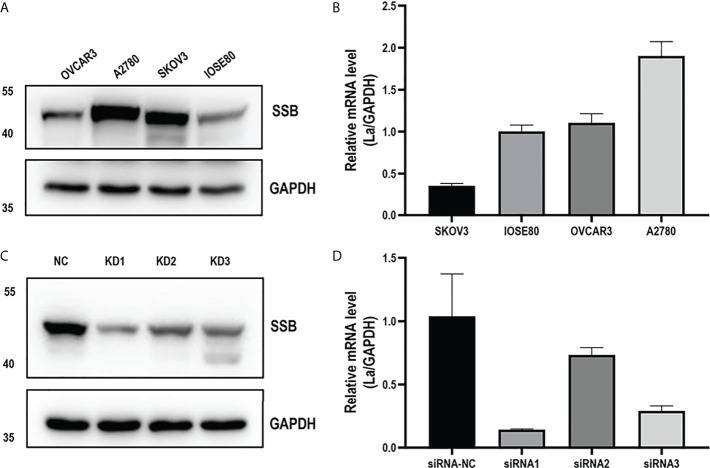
La mRNA level **(A)** and protein expression level **(B)** in ovarian cancer cells and human normal ovarian epithelial cells. The expression of La mRNA **(C)** and protein **(D)** in A2780 cells interfered by La lentivirus and control lentivirus. All data in the histogram are expressed as the mean ± standard deviation of three independent samples.

The expressions of La mRNA and protein in ovarian cancer cell lines A2780, OVCAR3, and SKOV3 and normal ovarian epithelial cell line IOSE-80 were detected. In these three ovarian cancer cell lines, the expression of La mRNA and protein in A2780 cells was higher than that in IOSE-80 cells, and its expression level was the highest among the four cell lines (**Figures 3A, B**). According to the La gene sequence, the interference sequence and a negative control sequence were designed. The lentiviral vector was constructed and packaged, and the lentivirus-mediated RNAi was performed on A2780 cells. After transfection, the expression of La was detected by Western blot and q-PCR. The interference efficiency of two interference groups (KD1 and KD3) was more than 70% compared with the control group (NC) (**Figures 3C, D**). Therefore, we chose KD1 and kD3 groups for the following experiments. KD1 and kD3 La lentiviruses were also used for RNA interference in SKOV3 cells (**Supplementary Figure 1**).

### Interference of La expression inhibits the viability of ovarian cancer cells

**Figure 4:**
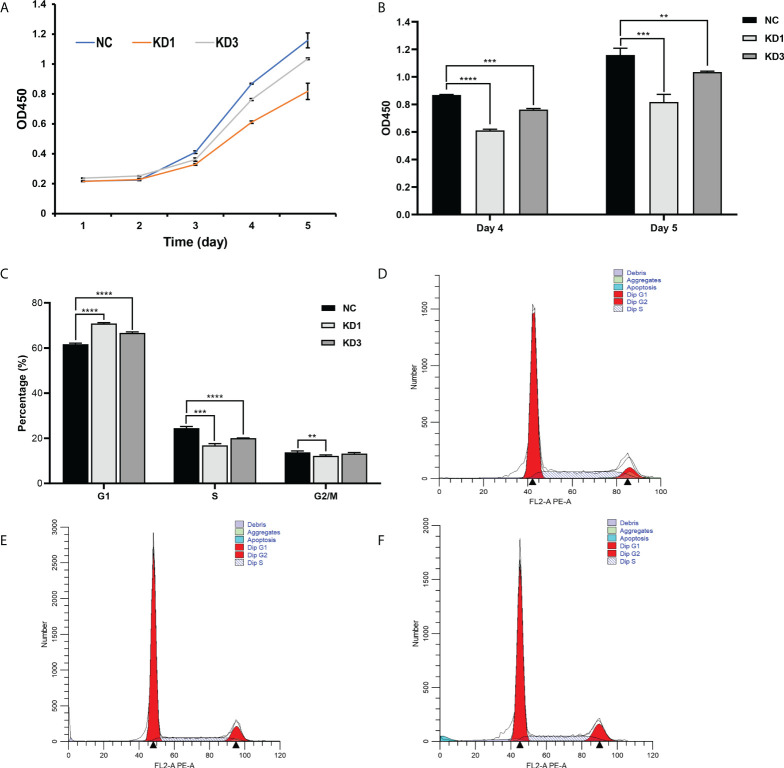
Effect of La RNAi on the viability and cell cycle of ovarian cancer A2780 cells. Cell viability kit was used to detect the effect of La RNAi on the viability of ovarian cancer A2780 cells **(A)**, and Cell Counting Kit-8 was used to detect the effect of La RNAi on the viability of ovarian cancer A2780 cells on the 4th and 5th days **(B)**. The effect of La RNAi on the cell cycle of ovarian cancer A2780 cells **(C)** in the NC group **(D)**, KD1 group **(E)**, and KD3 group **(F)**. All data were expressed as mean ± SD (^**^ *P* < 0.05, ^***^ *P* < 0.01, ^****^ *P* < 0.001).

CCK-8 was used to detect the OD450 of the three groups of cells for 5 consecutive days. It showed that the viability of KD1 and KD3 of A2780 cells treated with La RNAi was significantly downregulated compared with the control group (**Figure 4A**). On the 4th and 5th days, the viability of the two groups of cells treated with La RNAi was significantly reduced than that of the control group (**Figure 4B**). KD1, KD3, and NC groups were detected by flow cytometry under the same conditions. As shown in **Figures 4C–F**, the proportion of cells in the G1 phase in the KD1 group was significantly increased, while the proportion of cells in the S phase and G2/M phase was significantly decreased compared with the NC group. The proportion of cells in the G1 phase was significantly increased, and the proportion of cells in the S phase was significantly decreased in the KD3 group compared with the NC group. The same impaired cell growth associated with La interference was also observed in SKOV3 cells (**Supplementary Figure 2**).

### Interference of La expression promotes apoptosis of ovarian cancer cells

**Figure 5:**
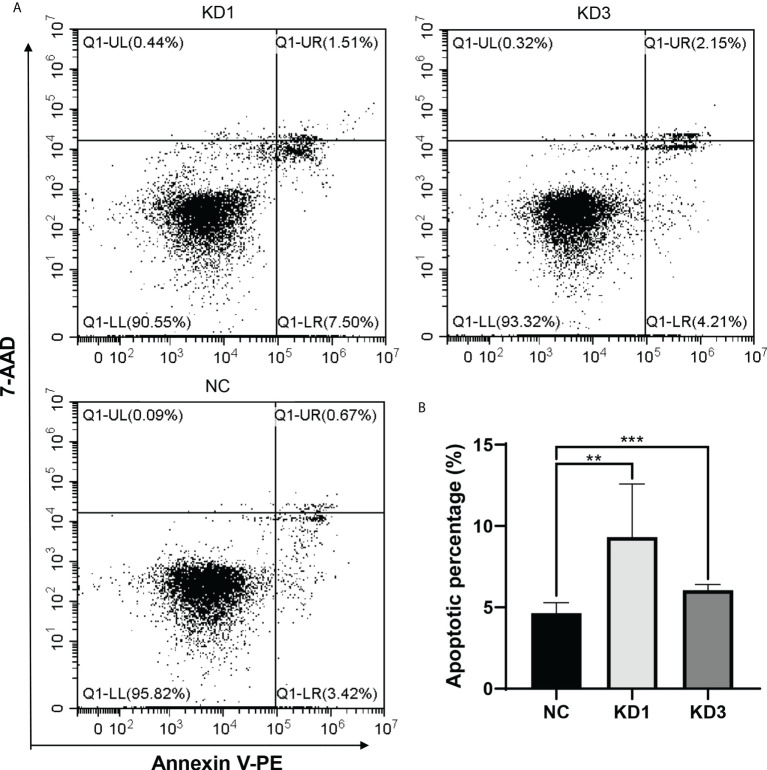
Effect of La RNAi on apoptosis of A2780 cells: **(A)**The apoptosis in A2780 cells of the KD1, KD3, and NC groups. **(B)** The percentage of apoptotic cells in the NC, KD1, and KD3 groups. The value was expressed as the mean ± standard deviation of three independent samples (^**^ *P* < 0.05, ^***^ *P* < 0.01).

Flow cytometry was used to detect the apoptosis of A2780 and SKOV3 cells of the KD1 and KD3 groups and the NC group. As shown in **Figure 5** and **Supplementary Figure 5**, interference of La led to significant increases in apoptosis of the KD1 and KD3 groups compared with the NC group. The result suggested that interference of La expression promotes apoptosis of ovarian cancer cells.

### Interference of La expression inhibits invasion and migration of ovarian cancer cells

**Figure 6:**
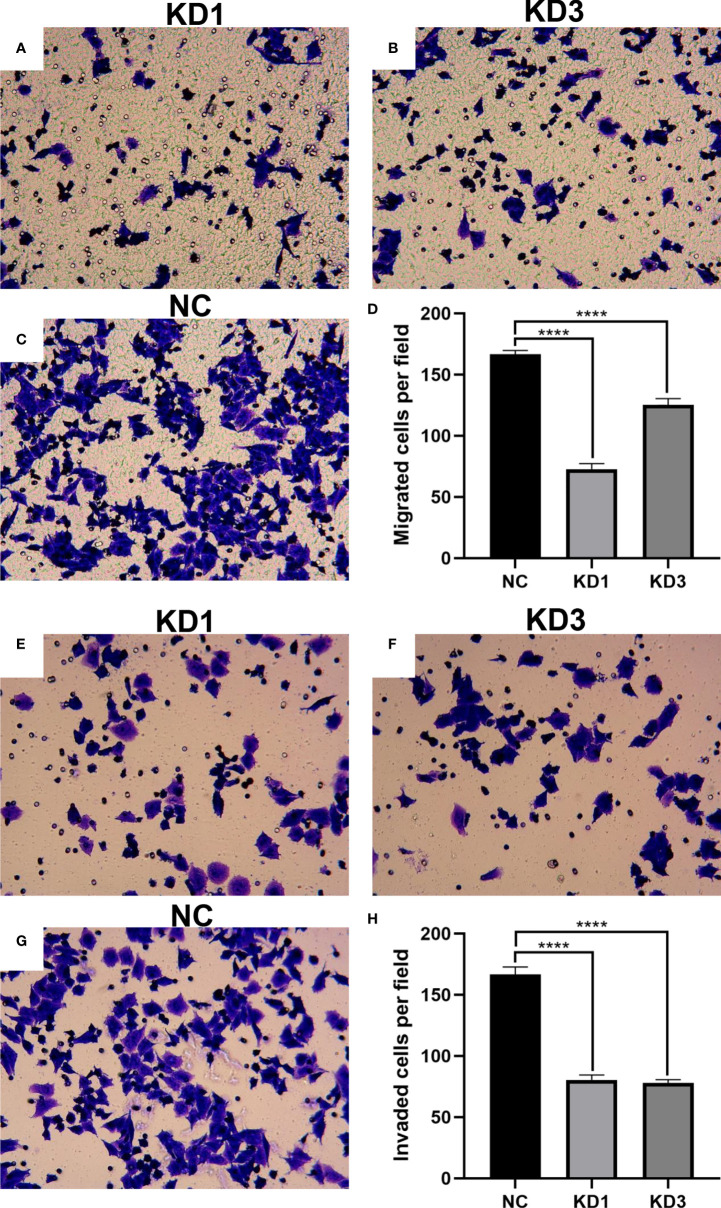
Effect of La RNAi on the migration and invasion of A2780 cells: migration chamber in the KD1 group **(A)**, KD3 group **(B)**, and NC group **(C)**, and the number of migration cells **(D)**. The effect of La RNAi on the invasion of A2780 cells: invasion chamber in the KD1 group **(E)**, KD3 group **(F)**, and NC group **(G)**, and number of metastatic cells in the invasion chamber **(H)**. All data were expressed as mean ± SD (^****^ *P* < 0.001).

Cell migration and invasion of KD1, KD3, and NC groups were conducted with Corning Inc. Transwell chambers and Matrigel invasion chambers. As shown in **Figures 6A–D**, the effect of La protein on A2780 cell migration was determined in the Transwell migration assay. The number of cells in the migration chambers of the KD1 and KD3 groups decreased significantly compared with the control group. The invasive behavior of the A2780 cells was explored by using the Transwell Matrigel invasion assay, which required the cells to invade through an extracellular Matrigel barrier. As shown in **Figures 6E–H**, La RNAi significantly decreased the invasive abilities of KD1 and KD3 groups compared with the NC group. Interference of La expression significantly inhibited the invasion and migration of A2780 cells as well as SKOV3 cells (**Supplementary Figures 3, 4**).

### Proteomic analysis of A2780 cells with the interference of La

**Figure 7:**
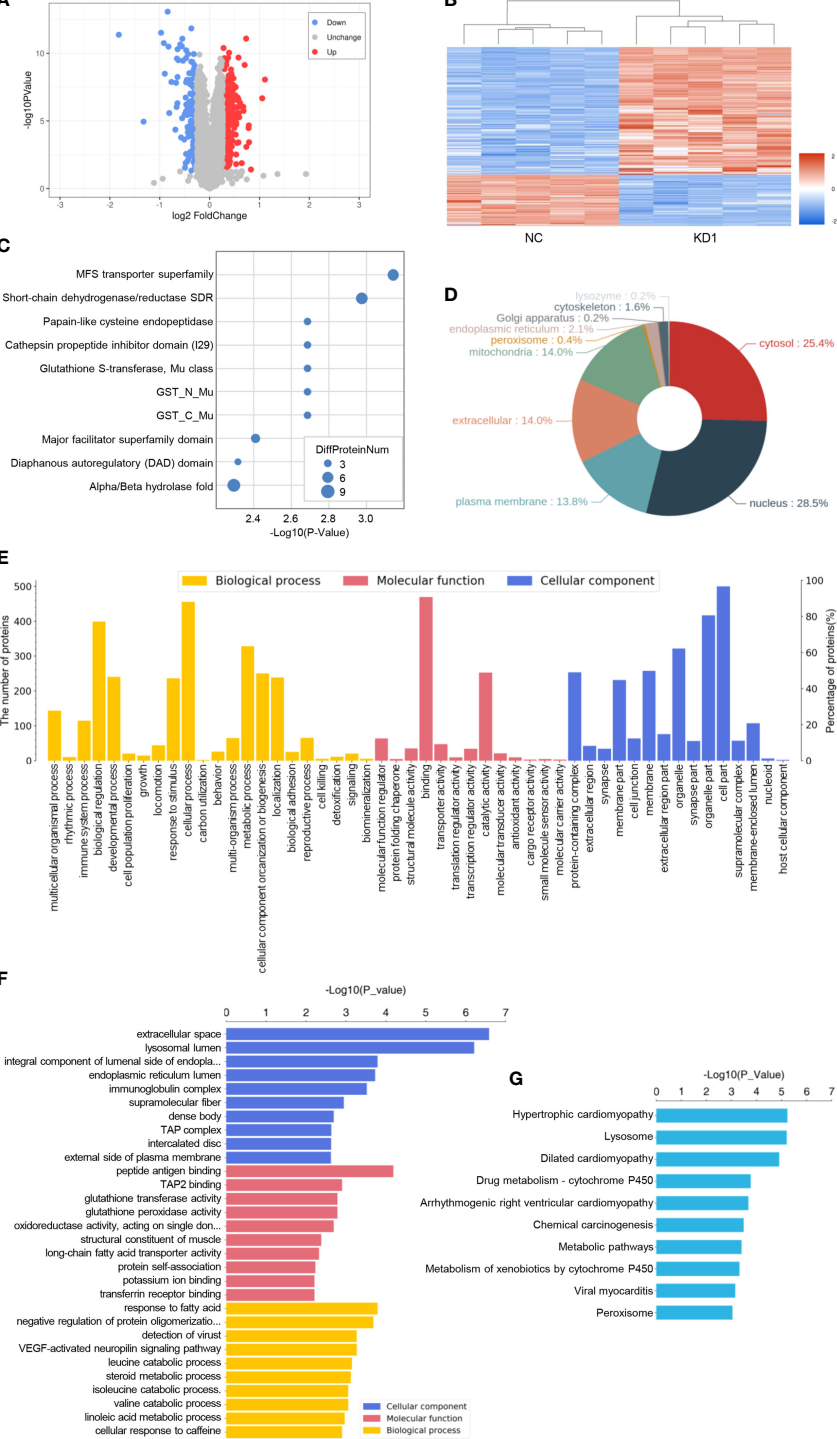
Proteomic analysis of A2780 cells with the interference of La. **(A)** The volcano map of differentially expressed proteins. The blue circle on the left represents the number of downregulated proteins, and the red circle on the right represents the number of upregulated proteins. Abscissa is the difference multiple (logarithmic transformation with 2 as the base). The ordinate is the value of *P* (logarithmic transformation based on 10). **(B)** Cluster analysis of differentially expressed proteins. **(C)** Statistical bubble chart (top 10) of protein domains with significant enrichment. **(D)** Protein subcellular localization map. **(E)** Level 2 statistical histogram of GO annotation. **(F)** The statistical histogram of the GO term with significant enrichment (top 10). The ordinate represents the name of Go function, and the abscissa represents the *P* value of enrichment significance. **(G)** Statistical histogram of the KEGG pathway with significant enrichment (top 10). The ordinate represents the name of the KEGG pathway, and the abscissa represents the *P* value of enrichment significance.

In order to research the effect of La RNA interference on the expression of proteins in ovarian cancer A2780 cells, proteomic analysis was done in the KD1 group and NC group with five biological replicates in each group. A total of 6,309 proteins were identified. According to the screening criteria of expression difference multiples greater than 1.2 times (up- or downregulation) and P value (t test) less than 0.05, 517 differentially expressed proteins were screened, of which 369 were downregulated and 148 were upregulated. The microarray data of differentially expressed proteins were shown in the volcano map (**Figure 7A**), and the heat map showed the clustering analysis results of protein expression in the La RNAi group and control group (**Figure 7B**). The classification of protein domains with significant enrichment is shown in the bubble chart (**Figure 7C**), and the differentially expressed proteins are mainly distributed in the nucleus (**Figure 7D**). GO analysis (**Figures 7E, F**) was used to determine the molecular functions, biological processes, and cellular components of the differentially expressed proteins. It showed that the differentially expressed proteins were involved in lysosomal cavity, immunoglobulin complex, histocompatibility complex, and other cell components. The main molecular functions of differentially expressed proteins included peptide antigen binding, transporter associated with antigen processing 2 (TAP2) binding, and other immune functions. The main biological functions of differentially expressed proteins included vascular endothelial growth factor (VEGF)-activated neuropilin signaling pathway, multiple amino acid catabolism, and negative regulation of protein oligomerization. KEGG enrichment analysis (**Figure 7G**) showed that the differentially expressed proteins were involved in chemical carcinogenesis, metabolic pathways, metabolism of xenobiotics by cytochrome P450, and so on.

### Identification of La-related RNAs in A2780 cells

**Figure 8:**
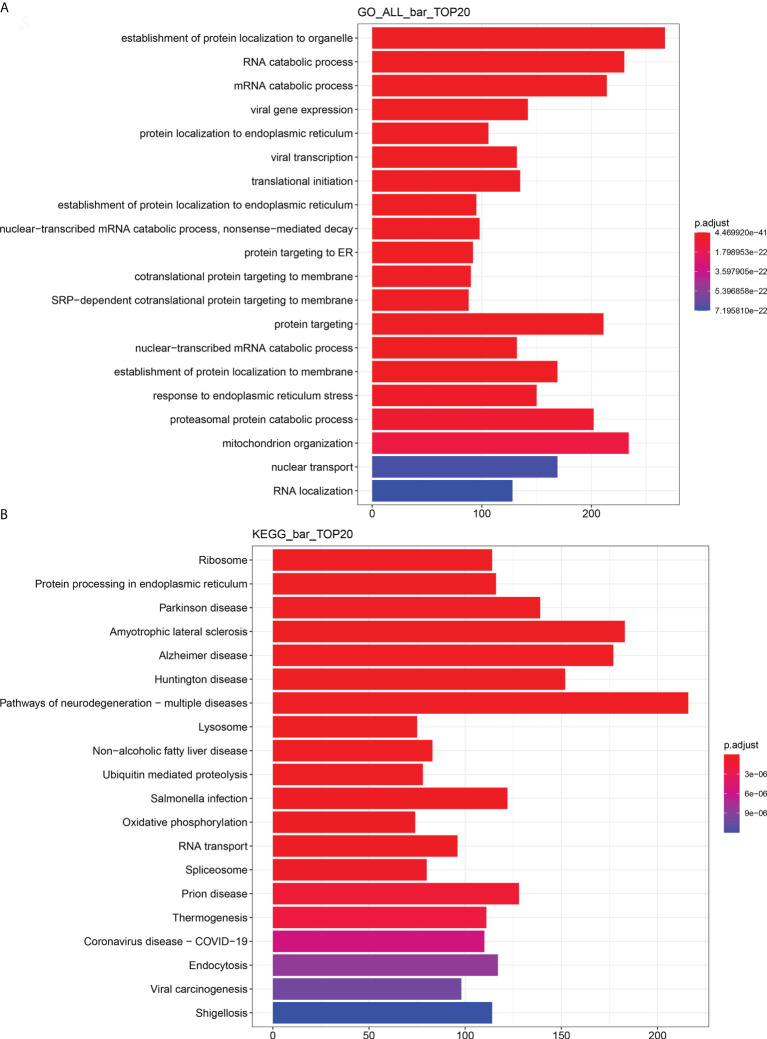
Enrichment analysis of RIP-seq GO and KEGG (top 20). **(A)** Total GO enrichment analysis. **(B)** Total KEGG enrichment analysis. The bar chart showed the top 20 items of enrichment degree.

In order to identify and characterize the La–mRNA complex in A2780 cells, La protein-specific antibody was used for RIP, library preparation, and sequencing of the total protein extract of exponential growth culture. As negative control, the protocol was carried out simultaneously with a non-specific IgG group. There were three groups of biological duplication. In A2780 cells, the RNAs binding with La protein were involved in endoplasmic reticulum stress, RNA transport, localization, metabolism, viral carcinogenesis, cell adhesion, and so on (**Figure 8** and **Supplementary Figures 6-8**).

### Prediction of downstream target of La protein in ovarian cancer

**Figure 9:**
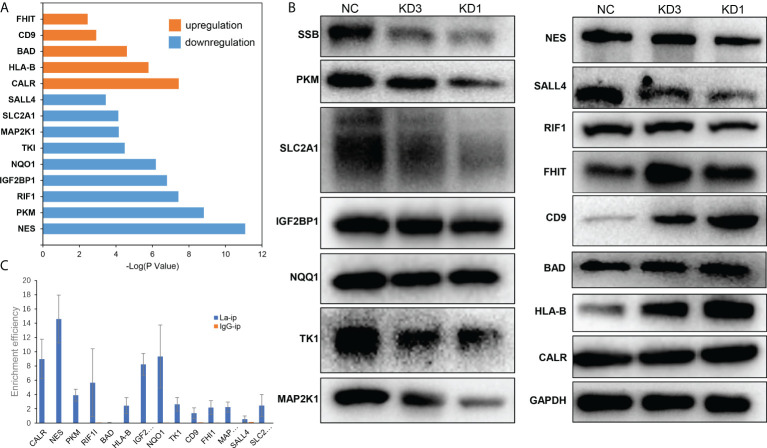
Possible downstream targets of La protein screened by RIP-seq and proteomics in A2780 cells. **(A)** The results showed that the relative expression of upregulated (orange) and downregulated (blue) proteins in A2780 cells after La RNA interference, and the ordinate was *P* value (logarithmic transformation with 10 as the base). **(B)** Western blot validation of La downstream targets. **(C)** RIP-qPCR validation of La binding to the selected mRNA.

The differentially expressed proteins were searched in literature to sort out the proteins that were significantly related to the development and drug resistance of ovarian cancer. Then an intersection was made between the mRNAs binding to La screened by RIP-seq and the genes related to La RNA interference screened by quantitative proteomics. Fourteen possible downstream target genes of La protein were screened out, which were NES, PKM, CALR, RIF1, IGF2BP1, NQO1, HLA-B, BAD, TK1, MAP2K1, SLC2A1, SALL4, CD9, and FHIT (**Figure 9A**). Then, Western blot and RIP-qPCR assays were conducted to verify La downstream targets identified through quantitative proteomics and RIP-RNA-seq. The results of quantitative proteomics and Western blot showed that La RNAi would affect the protein expression of these genes (**Figure 9B**). The results by RIP-seq and RIP-qPCR showed that La protein could bind to the mRNA of CALR, NES, PKM, RIF1, HLA-B, IGFRBP1, NQQ1, TK1, FHIT, MAP2K1, and SLC2A1 (**Figure 9C**). These proteins played an important role in the occurrence and development of ovarian cancer. The La protein might regulate the expression of these proteins to promote the proliferation, invasion, migration, and chemotherapy resistance of ovarian cancer and lead to poor prognosis of patients with ovarian cancer.

## Discussion

In the present study, we found that the expression of La protein in serum of patients with ovarian cancer was increased. Then the expression of La protein in ovarian cancer tissue was detected. The results suggested that La protein was abundantly expressed in ovarian cancer tissue and it was positively related to the malignant degree. The survival analysis of patients with ovarian cancer also showed that the high expression of La protein was related to the poor prognosis of platinum therapy for ovarian cancer, which further suggested the significance of La protein in ovarian cancer. Moreover, some studies have shown that La protein in cancer cells will migrate from the nucleus to cytoplasm under stress conditions and fix itself in the cytoplasm. It would be easily detected by a specific antibody. Therefore, La is feasible as a detection index (22). In the future, if a larger sample size of patients with ovarian cancer was detected for the La protein, richer data of La expression would be obtained, and a comprehensive statistical correlation analysis would be carried out with the pathological diagnosis and other biochemical tests of patients. It may help to form a new method for the detection of ovarian cancer.

Our present study suggested that the high expression of La protein in ovarian cancer A2780 and SKOV3 cells could promote the viability, invasion, and migration, inhibit apoptosis, and regulate the cell cycle to make A2780 cells in a state of high proliferation activity. La RNAi induced impaired cell growth to some degree in both A2780 and SKOV3 cells, which might be related to the regulation of G1/S-specific Cyclin D1 (CCND1) expression. As the RNA chaperone, La protein could bind to CCND1 mRNA, promote IRES-mediated translation, increase CCND1 expression, change the cell-cycle process, and enhance proliferation (23).

La RNAi downregulated the expression of NES, PKM, RIF1, IGF2BP1, NQO1, TK1, MAP2K1, SLC2A1, and SALL4 in A2780 cells. Nestin encoded by the NES gene is considered as a marker of angiogenesis and CSC in epithelial ovarian cancer and is associated with poor prognosis of various cancers (24). PKM2 increases the migration and invasion of ovarian cancer cells in lung metastasis *in vivo* (25). RIF1 promotes the growth and development of human epithelial ovarian cancer by activating the reverse transcriptase of telomerase (26). SRC is a membrane-associated non-receptor tyrosine kinase. It is highly expressed in most advanced tumor tissues, and it is an indicator of poor clinical prognosis (27). The SRC/mitogen-activated protein kinase (MAPK) signaling pathway drives the invasive growth of ovarian cancer cells. IGF2BP1 is an invasive growth driver of ovarian cancer targeting the SRC/MAPK signaling pathway (28). NQO1 has a protective effect on redox cycle, oxidative stress, and tumor formation, which is upregulated in ovarian cancer and significantly correlated with poor prognosis in patients with serous ovarian cancer (29). TK1 is an enzyme of the pyrimidine metabolic pathway involved in the rescue of DNA synthesis. The survival rate of patients with high TK1 gene expression was significantly lower than that of patients with low TK1 gene expression (30). High expression of MAP2K1 is significantly associated with the development of ovarian cancer and platinum resistance. Pimasertib, an inhibitor of MAP2K1, has been developed for the treatment of ovarian cancer (31). SLC2A1 mediates glycolysis, affects the tumor microenvironment, and affects the metabolism and metastasis of ovarian cancer cells (32). SALL4 is a marker of poor prognosis in serous ovarian cancer, which can promote the invasion and metastasis of ovarian cancer cells (33).

La RNAi could upregulate the expression of CALR, HLA-B, BAD, CD9, and FHIT in A2780 cells. The expression of CALR in primary ovarian tumors is associated with survival and response to chemotherapy in patients with ovarian cancer (34). CALR can activate the immune response of dying cancer cells, and the loss of CALR has proved to have a significant negative effect on the overall survival of patients with ovarian cancer (35). A decreased expression or deletion of HLA-B is associated with high invasiveness and increased malignant potential of ovarian cancer (36). Bad is a proapoptotic gene, which can promote the apoptosis of ovarian cancer cells and enhance the effect of chemotherapy (37). CD9 shows that the tumor grade was inversely proportional to its expression, which is characterized by high expression in low-grade tumors and low expression in high-grade tumors or metastatic tumors (38). FHIT is a tumor-suppressor gene, which is low expressed in most (possibly all) cancer types and can regulate the production of reactive oxygen species and apoptosis in cancer cells (39).

RIP results showed that La protein could bind to the mRNA of CALR, NES, PKM, RIF1, HLA-B, IGFRBP1, NQQ1, TK1, FHIT, MAP2K1, and SLC2A1. There have been sufficient studies to prove the RNA chaperone function of La protein, which is involved in all aspects of RNA metabolism. The biological function of La protein is based on its RNA-binding function (40). Therefore, it is suggested that La protein may play its RNA chaperone function by binding to the mRNA of the above 11 genes, regulating RNA transcription, translation, or stability, and further affecting protein expression. The role of La protein in the development of ovarian cancer may be regulating the abovementioned genes that have clear evidence to play important roles in ovarian cancer.

In summary, our study reveals a novel function of La protein whereby La protein plays its RNA chaperone function by binding with the mRNA of 11 genes and has a role in the pathological process of ovarian cancer. However, there are some limitations of this study: (1) Limited by the number of ovarian tissues detected, the current results are not enough to show the difference of La protein expression in different types of ovarian cancer tissues, such as endometrial adenocarcinoma and mucinous cystadenocarcinoma. A larger sample size will help to further explain the relationship between La expression and clinical features of ovarian cancer. (2) Whether the 11 genes are part of the downstream regulatory pathway of La protein in ovarian cancer needs to be further verified by targeted knockout, overexpression, or specific inhibitors of downstream target genes. Moreover, whether La protein can affect the transcriptional stability of the above genes needs to be further verified by transcriptional stability experiments with transcriptional inhibitors. (3) In addition, how La protein binds to the above mRNA and its specific binding mode, conformational change, and regulation mode need further research. Collectively, these results demonstrate a novel function and mechanism for La protein binding with the mRNA of 11 genes and having a role in the pathological process of ovarian cancer, which extends our understanding on La protein in terms of opening up novel therapeutic avenues in ovarian cancer.

## Data availability statement

The original contributions presented in the study are included in the article/**Supplementary Material**. Further inquiries can be directed to the corresponding author.

## Ethics statement

This study was reviewed and approved by the ethics committee of the Obstetrics and Gynecology Hospital of Fudan University (No. 2019-35). Written informed consent for participation was not required for this study in accordance with the national legislation and the institutional requirements.

## Author contributions

XH performed the research. JZ and XH wrote the manuscript. YL and YY corrected the writing and JT obtained funding, designed the research plan, and edited the manuscript. All authors contributed to the article and approved the submitted version.

## Funding

The work reported herein was supported by the grants from the Key Specialized Construction Projects of Clinical Pharmacy of Shanghai (AB83110002017005) and Oncology Pharmaceutical Research Fund Project of Shanghai Pharmaceutical Association (2020-01-03).

## Conflict of interest

The authors declare that the research was conducted in the absence of any commercial or financial relationships that could be construed as a potential conflict of interest.

## Publisher’s Note

All claims expressed in this article are solely those of the authors and do not necessarily represent those of their affiliated organizations, or those of the publisher, the editors and the reviewers. Any product that may be evaluated in this article, or claim that may be made by its manufacturer, is not guaranteed or endorsed by the publisher.
