# Supplementary material for: La protein regulates protein expression by binding with the mRNAs of target genes and participates the pathological process of ovarian cancer
Source: Front Oncol. 2022 Aug 30;12:763480. doi: 10.3389/fonc.2022.763480 (PMC9468491; doi:10.3389/fonc.2022.763480)
Supplement: Supplementary file 1 [file DataSheet_1.docx]

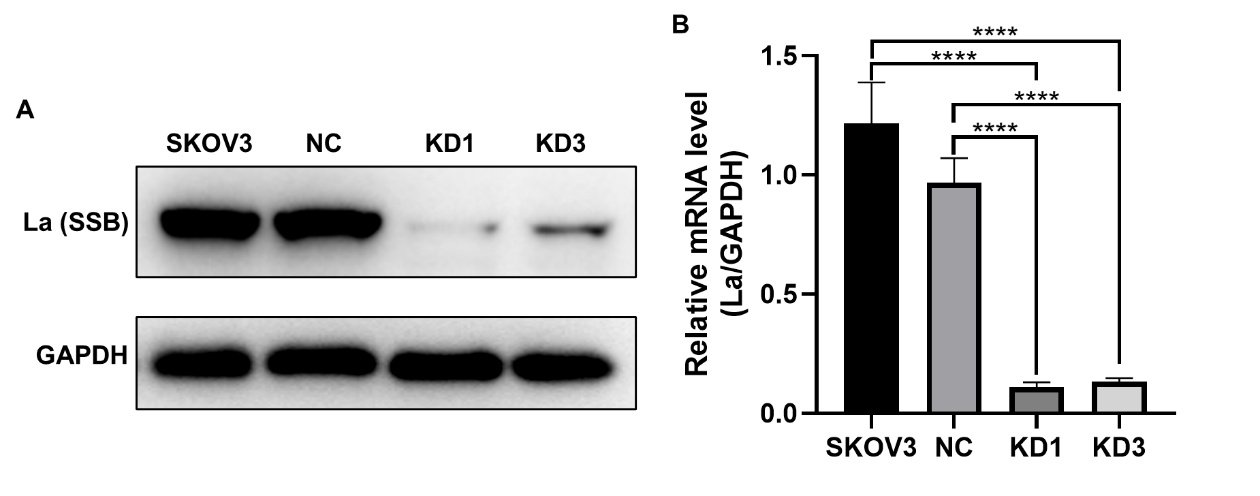


Supplementary Figure 1. The expression of La protein **(A)** and mRNA **(B)** in SKOV3 cells and SKOV3 cells interfered by La lentivirus KD1 and KD3 and control lentivirus. All data in the histogram are expressed as the mean ± standard deviation of three independent samples (^****^*P* < 0.0001).


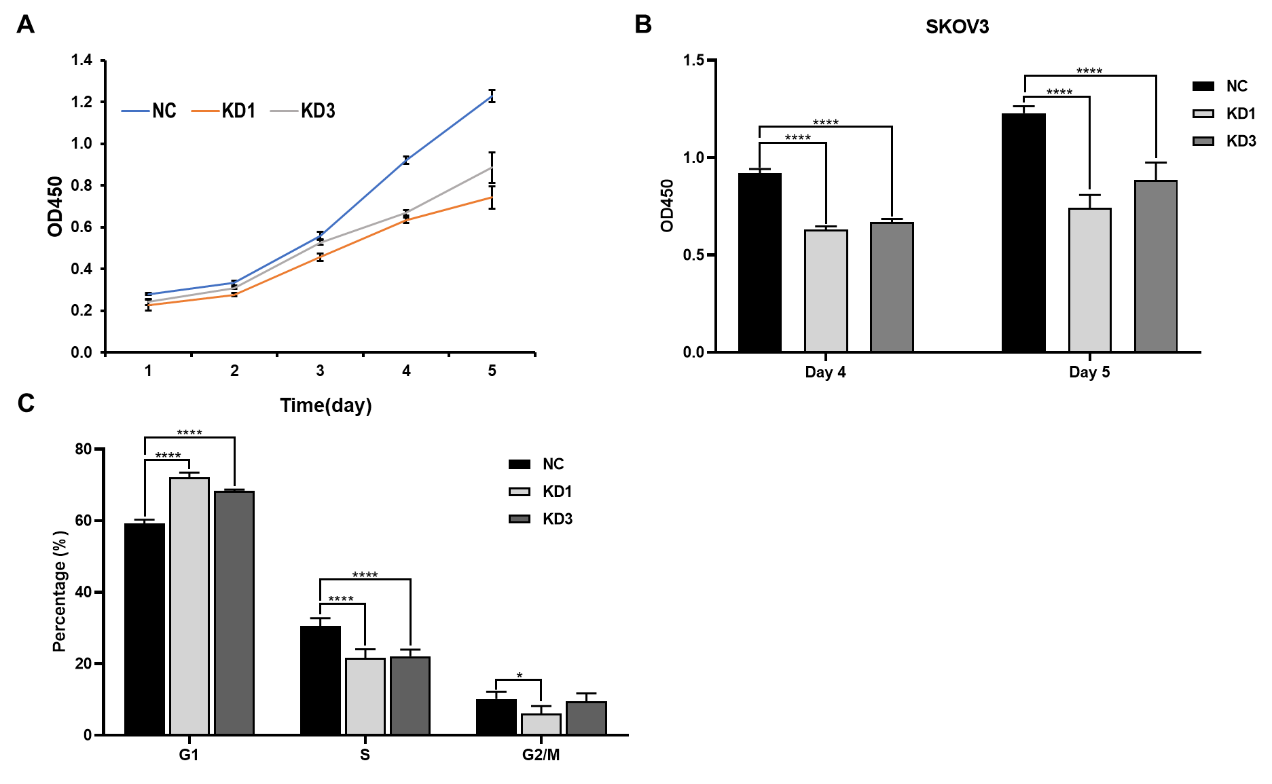


Supplementary Figure 2. Effect of La RNAi on the viability and cell cycle of ovarian cancer SKOV3 cells. Cell viability kit was used to detect the effect of La RNAi on the daily proliferation of ovarian cancer SKOV3 cells **(A)**, cell counting kit-8 was used to detect the effect of La RNAi on the viability of ovarian cancer SKOV3 cells on the 4th and 5th day **(B)**, and the effect of La RNAi on the cell cycle of ovarian cancer A2780 cells **(C)**. All data were expressed as mean ± SD (^*^*P* < 0.05, ^****^*P* < 0.0001).


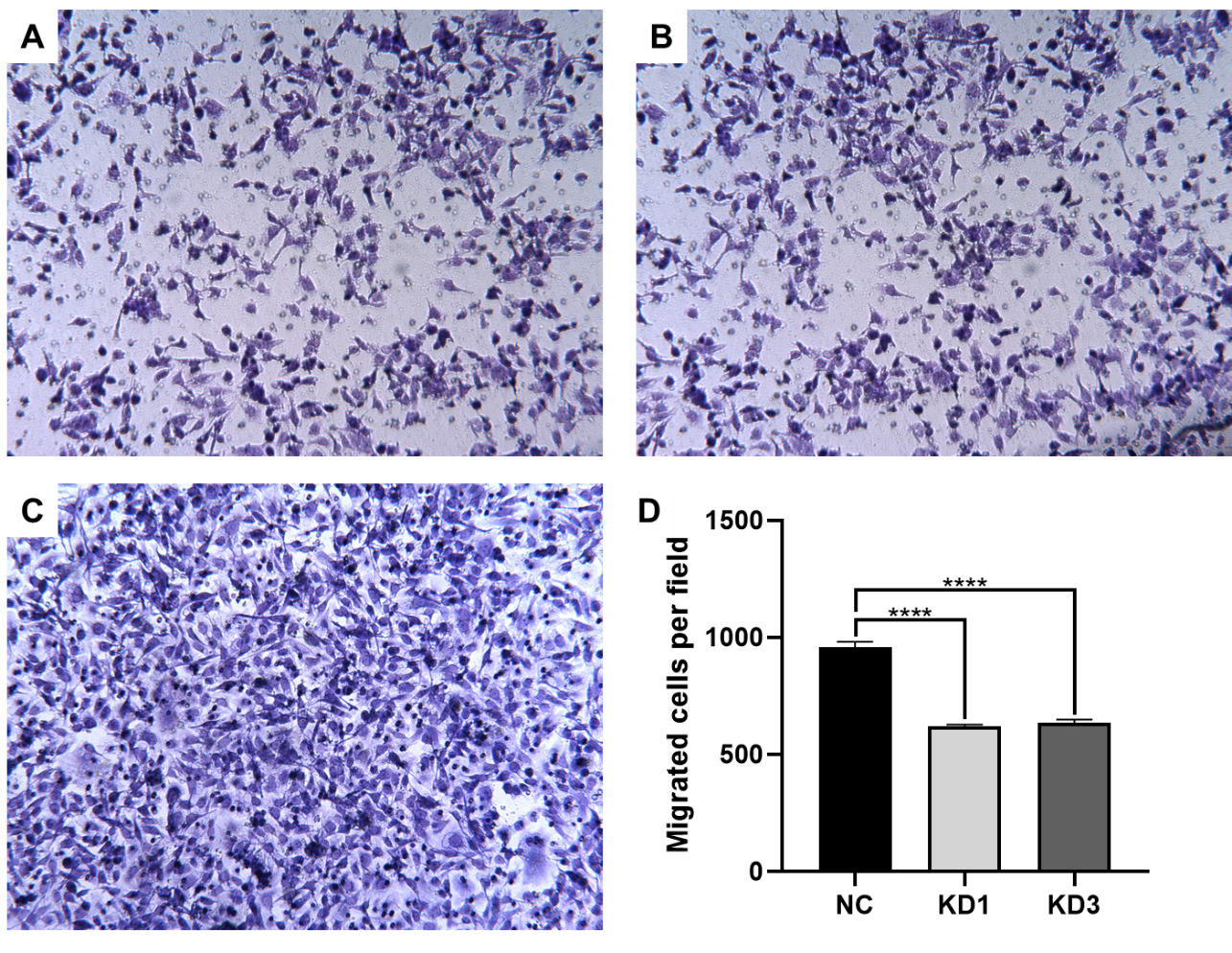


Supplementary Figure 3. Effect of La RNAi on the migration of SKOV3 cells: migration chamber in KD1 group **(A)**, KD3 group **(B)**, NC group **(C)**, and the number of migration cells **(D)**. All data were expressed as mean ± SD (^****^*P*<0.0001).


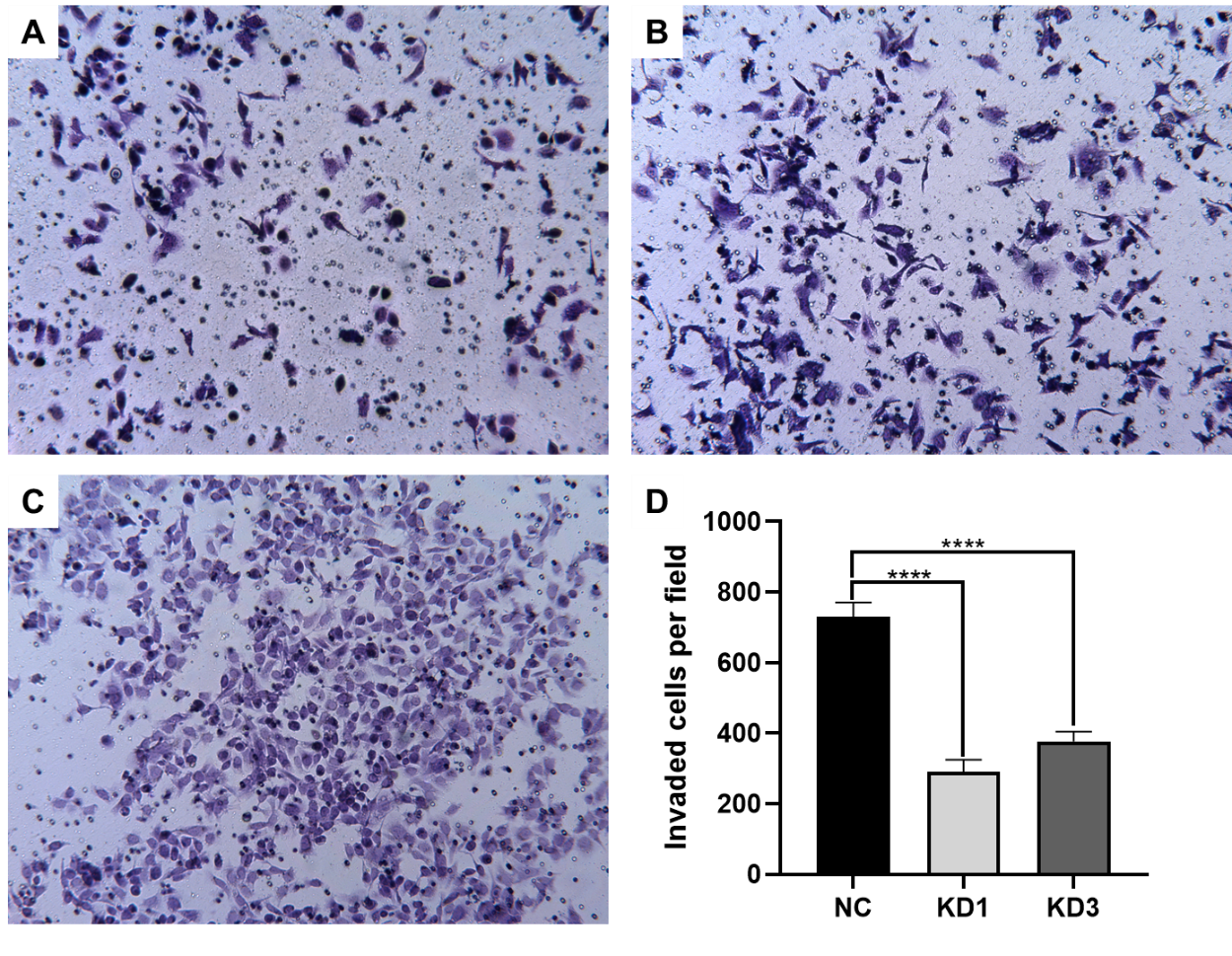


Supplementary Figure 4. Effect of La RNAi on the invasion of SKOV3 cells: invasion chamber in KD1 group **(A)**, KD3 group **(B)**, NC group **(C)**, and number of metastatic cells in invasion chamber **(D)**. All data were expressed as mean ± SD (^****^*P*<0.0001).


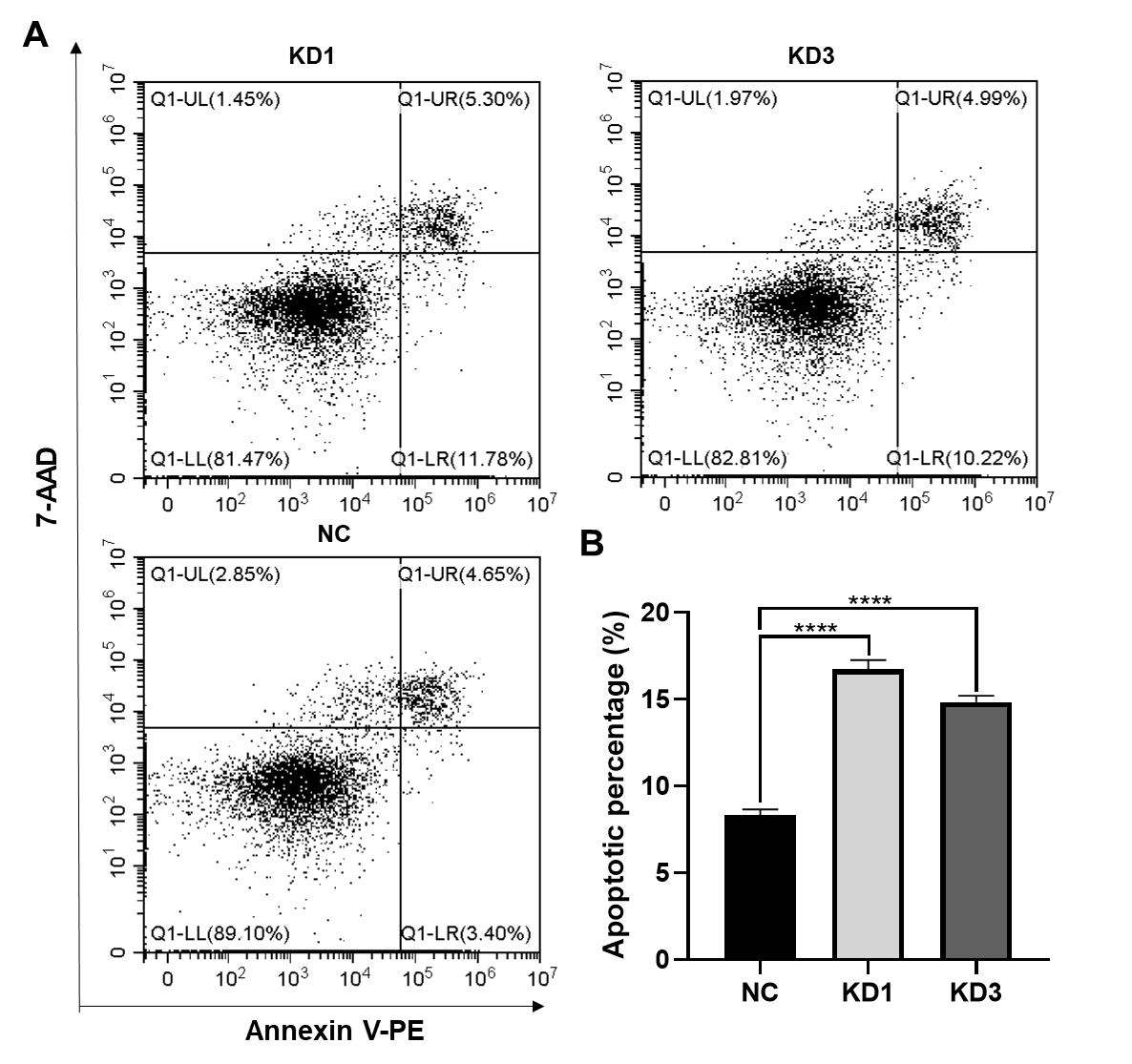


Supplementary Figure 5. Effect of La RNAi on apoptosis of SKOV3 cells: **(A)** The apoptosis in A2780 cells of KD1, KD3 and NC group, **(B)** The percentage of apoptotic cells in NC, KD1 and KD3 groups. The value was expressed as the mean ± standard deviation of three independent samples (^****^*P* < 0.0001).


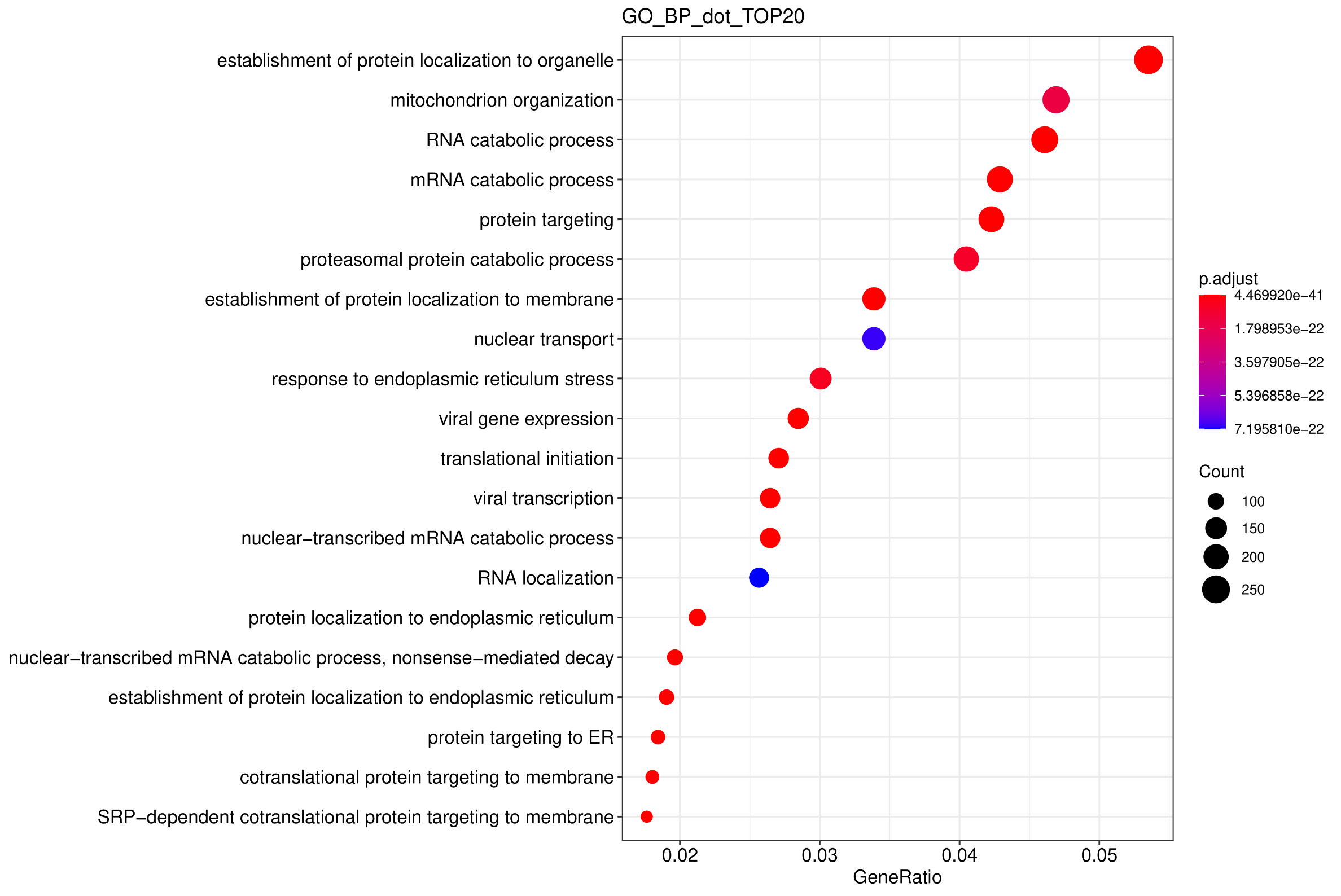

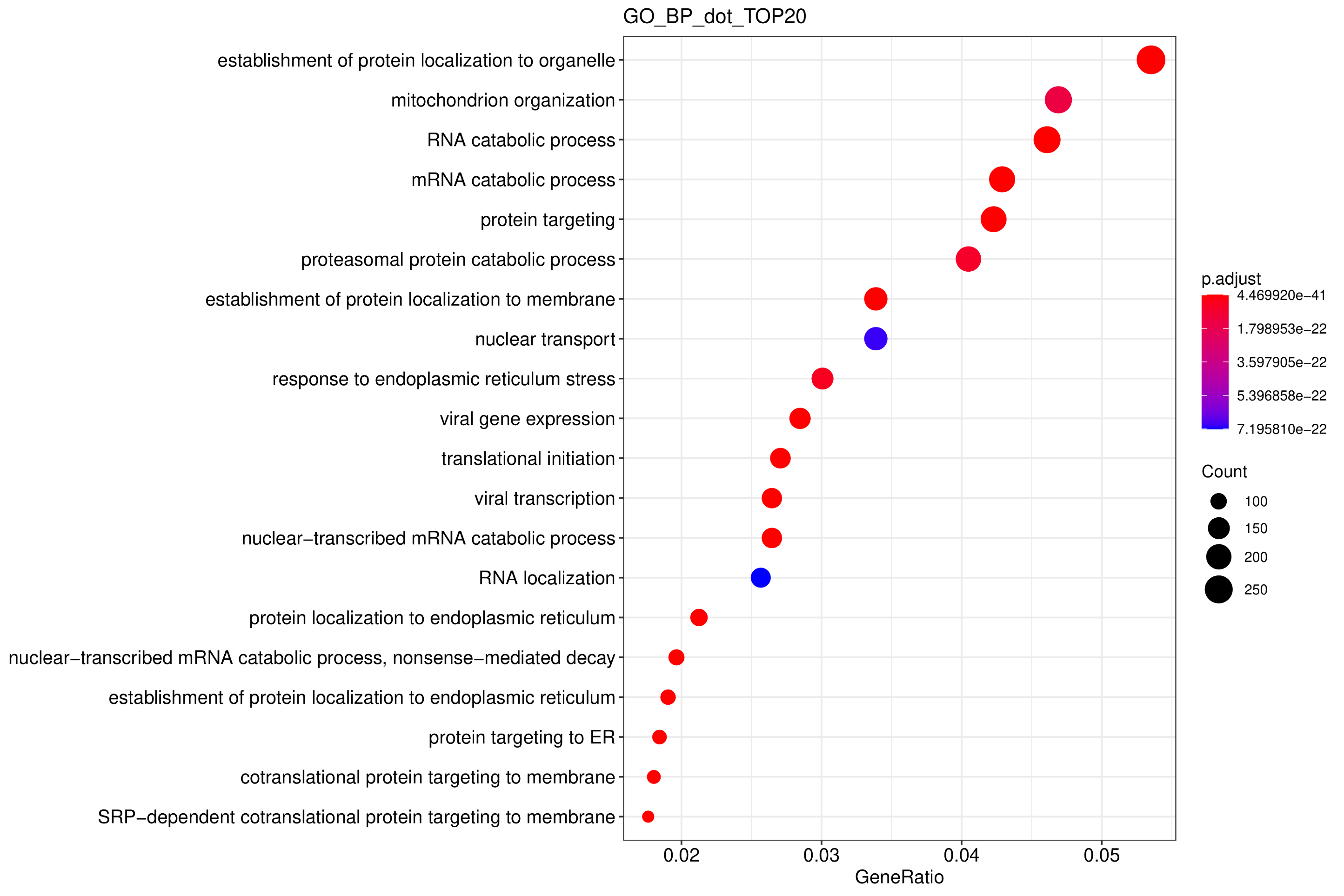

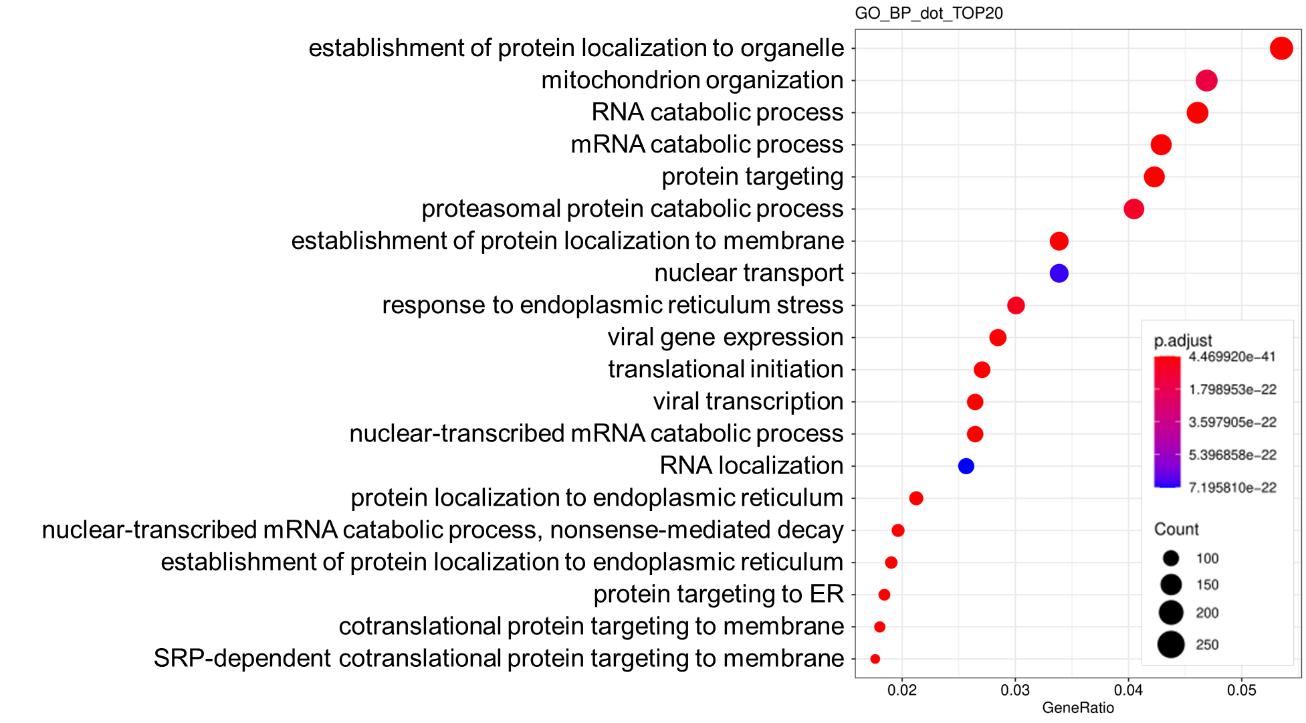


Supplementary Figure 6. Go enrichment analysis of biological process (top 20). BP, Biological Process. The bubble chart showed the top 20 items of enrichment degree, and the bubble size indicated the number of enriched differential proteins in the item.


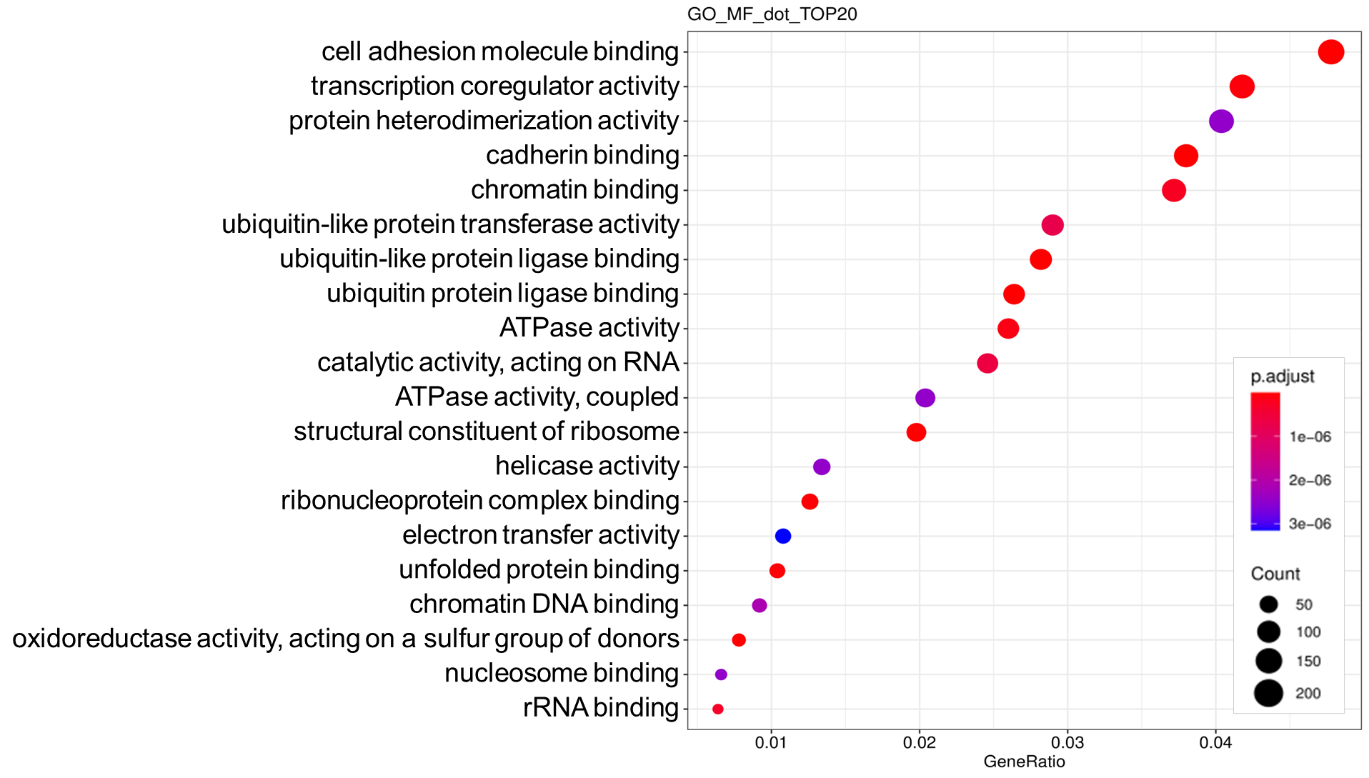


Supplementary Figure 7. Go enrichment analysis of molecular function (top 20). MF, Molecular Function. The bubble chart showed the top 20 items of enrichment degree, and the bubble size indicated the number of enriched differential proteins in the item.


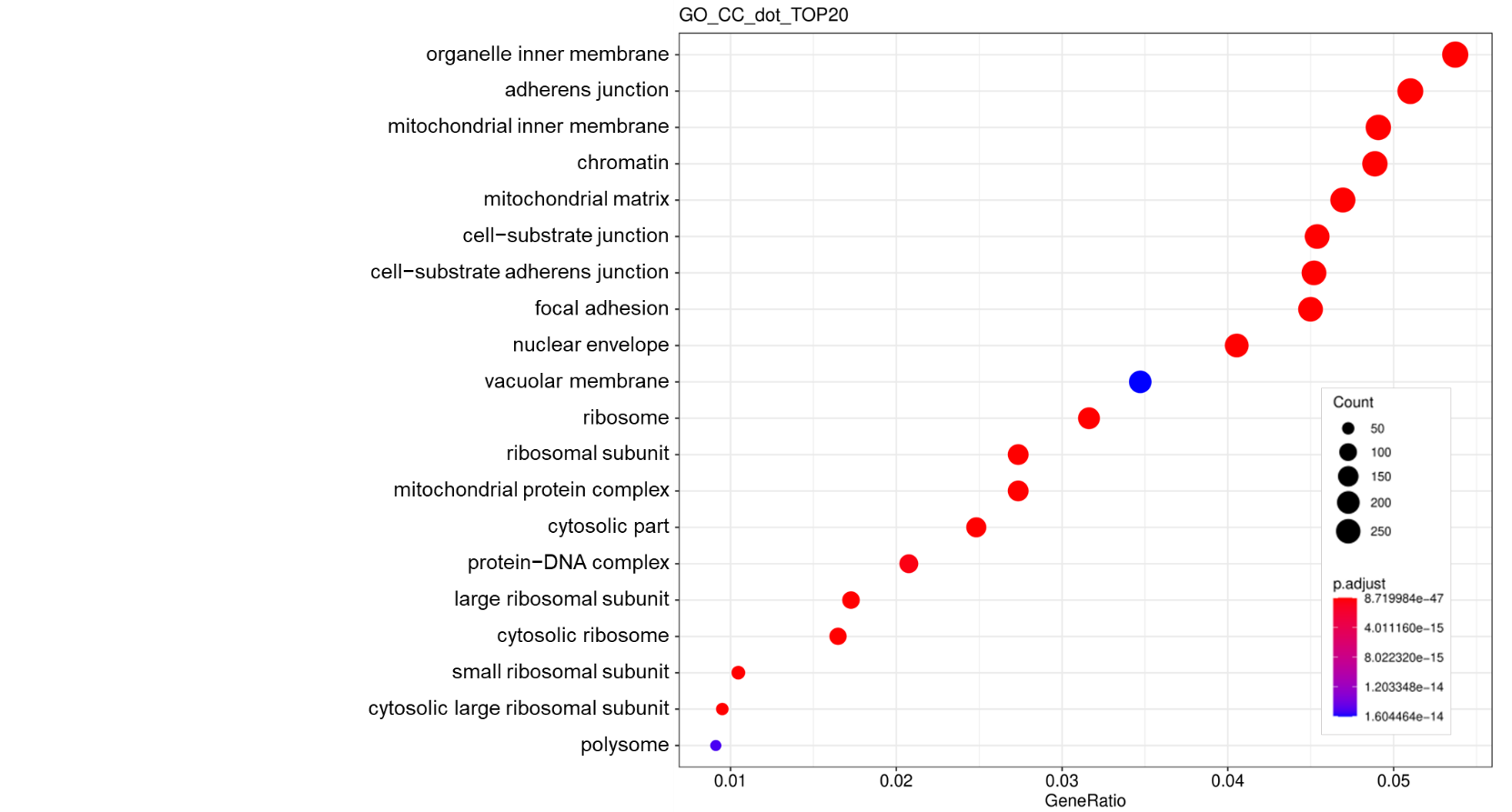


Supplementary Figure 8. Go enrichment analysis of cellular component (top 20). CC, Cellular Component. The bubble chart showed the top 20 items of enrichment degree, and the bubble size indicated the number of enriched differential proteins in the item.


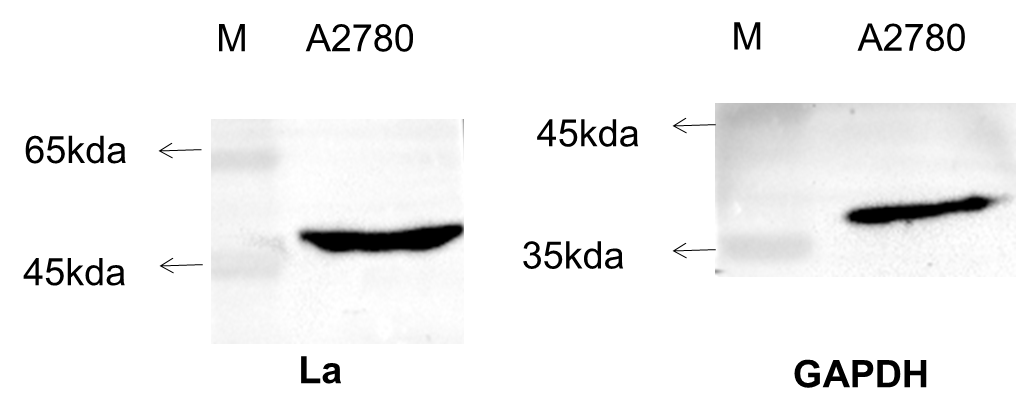


Supplementary Figure 9. Western blot data for the specific La immunoprecipitation critically for the La-RIP
